# La integración de la prueba de ctDNA en el análisis de *EGFR *en el carcinoma pulmonar no microcítico: estrategias para el laboratorio clínico

**DOI:** 10.1515/almed-2025-0092

**Published:** 2025-08-28

**Authors:** Esther Fernández-Galán, Joan Anton Puig-Butillé

**Affiliations:** Servicio de Bioquímica y Genética Molecular, 16493 Biomedical Diagnostic Center (CDB), Hospital Clinic de Barcelona, Barcelona, España; Fundació de Recerca Clínic Barcelona-Institut d’Investigacions Biomèdiques August Pi i Sunyer (IDIBAPS), Universidad de Barcelona, Barcelona, España; CORE de Biología Molecular, Centro de Diagnóstico Biomédico (CDB), Hospital Clínic de Barcelona, Barcelona, Spain; Servicio de Bioquímica y Genética Molecular, 16493 Biomedical Diagnostic Center (CDB), Hospital Clinic de Barcelona, Barcelona, Spain

**Keywords:** *EGFR*, biopsia líquida, ctDNA, cáncer de pulmón, marcadores tumorales séricos

## Abstract

El análisis molecular del gen receptor del factor de crecimiento epidérmico (*EGFR*) resulta fundamental a la hora de seleccionar una terapia dirigida para el carcinoma pulmonar no microcítico avanzado (CPNM). Entre el 15 % y el 40 % de los pacientes con CPNM son portadores de mutaciones en *EGFR* sensibles a los inhibidores de la tirosina quinasa (TKI, por sus siglas en inglés). Para optimizar el tratamiento, resulta esencial poder identificar a los pacientes con mayor probabilidad de obtener un beneficio clínico de la terapia con TKI. Aunque el análisis del gen *EGFR* se suele realizar en tejido tumoral, no se puede obtener material suficiente que permita realizar esta prueba en hasta un 30 % de los pacientes. En este contexto, las guías de práctica clínica recomiendan realizar un análisis de ADN tumoral circulante (ctDNA, por sus siglas en inglés), como método no invasivo para la detección de mutaciones en *EGFR* Si bien se han obtenido resultados alentadores, el análisis de ctDNA presenta algunas limitaciones que dificultan su uso generalizado en la práctica clínica. Realizamos una revisión de los estudios realizados sobre la utilidad clínica del análisis molecular del *EGFR* en ctDNA. Así mismo, se analiza su relación con otros biomarcadores de uso frecuente en el laboratorio clínico, como los marcadores tumorales séricos (MTS) y se abordan algunos aspectos técnicos, o relacionados con la interpretación de resultados y otras dificultades asociadas al análisis de ctDNA, ofreciendo información valiosa para su integración en los flujos de trabajo del laboratorio.

## Introducción

La oncología de precisión ha influido profundamente en el manejo del cáncer de pulmón (CP), identificado como el tipo de cáncer más frecuente en 2022, con cerca de 2,5 millones de nuevos casos diagnosticados en todo el mundo, siendo además la principal causa de mortalidad por cáncer [1]. La identificación de los mecanismos moleculares implicados en el CP ha permitido desarrollar e implementar terapias dirigidas, especialmente para el carcinoma pulmonar no microcítico (CPNM).

El gen del receptor del factor de crecimiento epidérmico (*EGFR*) representa el oncogén accionable más prevalente y el primero en ser identificado como diana terapéutica en el CPNM, presentando entre el 15 y el 40 % de los pacientes mutaciones en *EGFR*. Los inhibidores de la tirosina quinasa EGFR (EGFR-TKI) se han convertido en la primera línea de tratamiento para los pacientes con mutaciones activadoras de EGFR, dado que con ellos se ha logrado aumentar la supervivencia y mejorar la calidad de vida de los pacientes [2]. De este modo, ante un diagnóstico de CPNM, resulta imperativo realizar un análisis molecular, con el objeto de identificar a los pacientes que puedan obtener un mayor beneficio clínico de la terapia con EGFR-TKI [3].

No obstante, la aplicación de estas pruebas en la práctica clínica habitual se ve obstaculizada por la dificultad a la hora de obtener tejido tumoral adecuado, que es la pieza angular de los estudios moleculares. Así, en hasta el 30 % de los pacientes con CP no se dispone de material suficiente para poder realizar el análisis molecular del gen *EGFR* [4]. Además, debido a la naturaleza invasiva de las biopsias, realizar un seguimiento continuado resulta inviable. Ante este escenario, se han desarrollado técnicas de “biopsia líquida” (BL), un método no invasivo, o mínimamente invasivo, para analizar las características del tumor en fluidos biológicos como la sangre [5]. Estos métodos detectan analitos derivados del tumor, como el ADN tumoral circulante (ctDNA), las células tumorales circulantes, el ARN libre circulante, y las vesículas extracelulares.

La presente revisión se centra principalmente en el análisis de ctDNA, una prueba sobradamente validada y de gran aceptación en la clínica. El análisis de mutaciones en *EGFR* en ctDNA es un método aceptado para identificar a los pacientes que se pueden beneficiar de la terapia con EGFR-TKI. Las pruebas de ctDNA son especialmente valiosas cuando no se dispone de muestras de tejido, o cuando es preciso un tiempo rápido de respuesta (TDR) para tomar decisiones terapéuticas urgentes. No obstante, aún es necesario resolver algunas dificultades que impiden la realización sistemática de análisis de ctDNA en el laboratorio clínico. Los profesionales del laboratorio desempeñan un papel fundamental a la hora de garantizar la fiabilidad de los resultados de estas pruebas y realizar una interpretación óptima de sus resultados, lo que les sitúa en una posición relevante a la hora de lograr su integración eficaz en la práctica clínica.

En esta revisión, se resume de forma exhaustiva el conocimiento existente sobre la aplicación clínica del análisis molecular del *EGFR* en el ctDNA. Desde el punto de vista del laboratorio clínico, exploramos los aspectos técnicos, las aplicaciones clínicas, y las ventajas y dificultades que esta prueba entraña, ofreciendo así mismo algunas recomendaciones prácticas para integrar eficazmente las pruebas de ctDNA en la práctica habitual.

## EGFR: estructura, señalización e implicaciones clínicas

La EGFR es una glucoproteína de transmembrana de 170-kDa perteneciente a una familia de cuatro receptores con un dominio en la superficie celular: (EGFR o HER1), HER2, HER3 y HER4. Los primeros estudios sobre la vía de señalización de EGFR se realizaron tras el descubrimiento del factor de crecimiento epidérmico (EGF) por parte de Cohen y Levi-Montalcini in 1962 [6], [7]. Desde entonces, los diversos estudios bioquímicos y genéticos llevados a cabo han permitido dilucidar su papel en la regulación del crecimiento celular, así como detallar la intrincada cascada de señalización intracelular que se inicia con la interacción ligando-receptor.

La oncoproteína EGFR se compone de tres dominios: un dominio extracelular rico en hidratos de carbono que facilitan la unión al ligando; un dominio hidrofóbico de transmembrana; y un dominio citoplasmático con actividad tirosinquinasa donde se encuentran los lugares de fosforilación. Ligandos como el EGF y el factor de crecimiento transformante (TGFα) se pueden unir a la EGFR. Al producirse la unión al ligando, la EGFR induce la dimerización con HER2 u otros miembros de la familia, formando homodímeros y heterodímeros. Esta dimerización facilita la autofosforilación, activando así la señalización descendente *(downstream signaling)* [8]. La activación de dicha cascada induce la proliferación celular, desempeñando un papel relevante en la supervivencia y diferenciación celular.

Por tanto, la sobreexpresión o activación inadecuada de EGFR podría contribuir al desarrollo y progresión del cáncer. La señalización de EGFR puede verse aumentada por la amplificación del gen *EGFR*, la sobreexpresión de proteínas, o la presencia de mutaciones activadoras específicas. El EGFR se sobreexpresa en tumores sólidos como en el cáncer de pulmón, cabeza y cuello, mama, riñón, colon, ovario, próstata, cerebro y vejiga [9]. Dicha sobreexpresión se ha asociado a una mayor agresividad del tumor y a una peor respuesta a las terapias convencionales.

**Figura 1:**
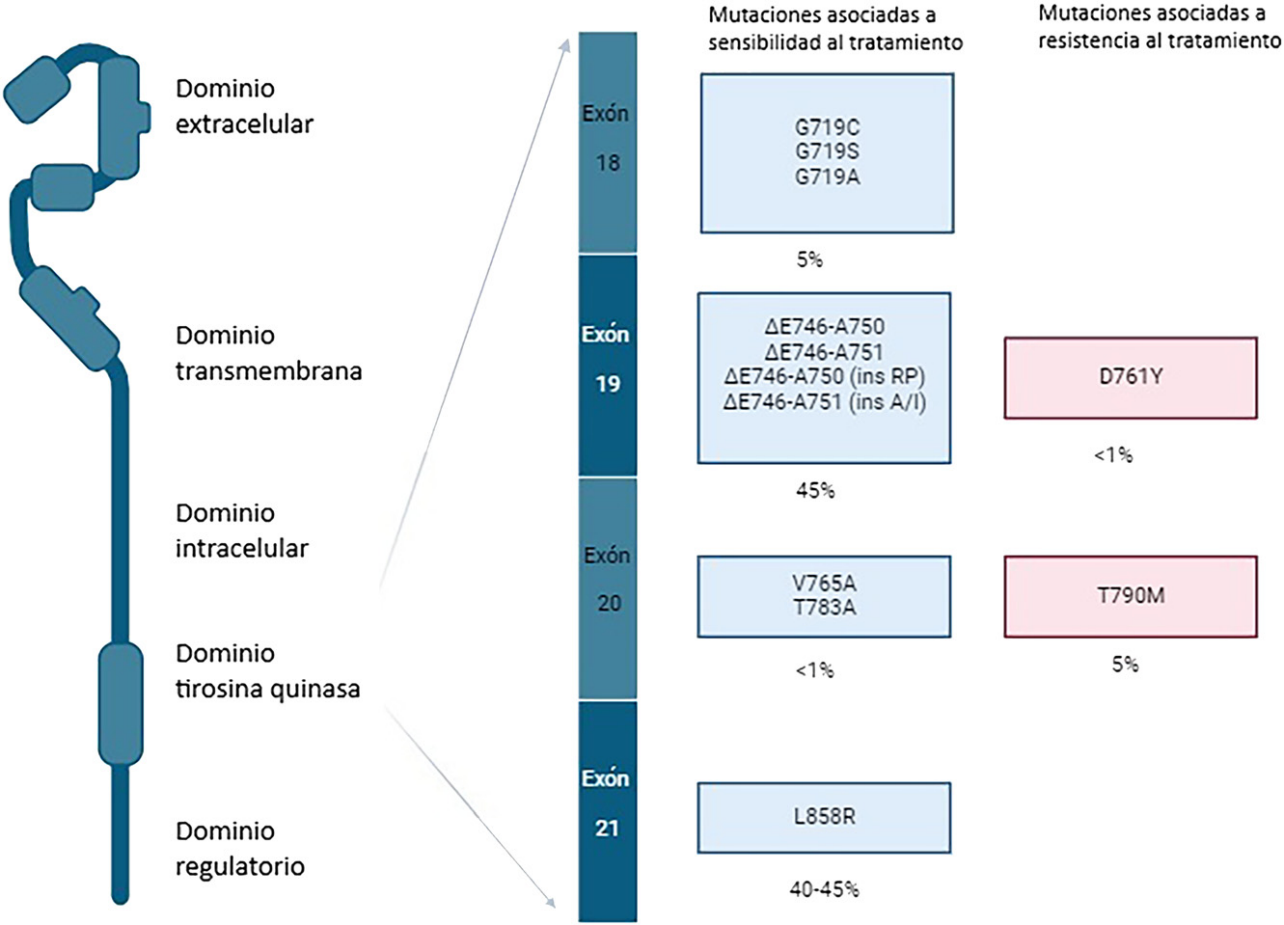
Relevancia clínica de las principales mutaciones en EGFR clave en el cáncer de pulmón no microcítico. En la Figura se muestra la relevancia clínica de las mutaciones en EGFR clave en el cancer de pulmón no microcítico (CPNM). Las mutaciones se muestran agrupadas según su localización en exones concretos (18–21) y su asociación con la sensibilidad o resistencia al tratamiento. Las mutaciones mostradas en los recuadros azules están relacionadas con una mayor sensibilidad a la terapia con inhibidores de la tirosina quinasa EGFR (TKI); también se muestran sus correspondientes porcentajes de prevalencia. Las mutaciones en los recuadros rojos son las relacionadas con la resistencia al tratamiento. Las deleciones en el exón 19 y la mutación L858R en el exón 21 son las mutaciones de sensibilidad al tratamiento más prevalentes, siendo, no obstante, la mutación T790M en el exón 20 la mutación de resistencia más frecuente. Así mismo, para proporcionar un contexto estructural, se muestran los dominios de la proteína EGFR (extracellular, de transmembrana, intracellular, tirosina quinasa y regulatorio). Adaptado de: Sharma SV et al.: Epidermal growth factor receptor mutations in lung cancer, *Nat Rev Cancer* 7: 169–181, 2007 y Bai Y, et al.: Molecular genetics of solid tumors, en Henry’s Clinical Diagnosis and Management by Laboratory Methods, 24ª ed., pp. 1579–1587, Elsevier, 2022. Figura generada con Biorender.

En el CPNM, la desregulación del EGFR es un fenómeno frecuente, con una sobreexpresión de proteínas en el 85 % de los casos, mutaciones genéticas en entre el 10 y el 40 % de los pacientes, y amplificación de genes en aproximadamente el 10 % de los casos. La sobreexpresión de proteínas (que se evalúa mediante inmunohistoquímica), o el número de copias de los genes (que se analiza mediante hibridación fluorescente *in situ)*, carecen de un valor pronóstico o predictivo consistente. Por otro lado, se ha demostrado que la presencia de mutaciones en *EGFR* es un factor predictivo de la respuesta a la terapia con EGFR-TKIs [10]. Estas mutaciones somáticas, que producen la activación constitutiva de la quinasa de EGFR, son más frecuentes en mujeres, en los pacientes con adenocarcinoma, de raza asiática, y en aquellos que nunca han tenido hábito tabáquico. Las mutaciones activadoras del gen *EGFR* más frecuentes en los casos de NSCLC son las deleciones en el exón 19 (>50 %), seguidas de la mutación p.L858R en el exón 21 (∼40 %), estando estas últimas asociadas a una respuesta positiva a la terapia con EGFR-TKI. El 10 % restante de las mutaciones del *EGFR* en el NSCLC son mutaciones en los exones 18 y 20 [11] (Figura 1).

La mutación puntual p.T790M es el mecanismo más común de resistencia adquirida, ya que se identifica en entre el 50 y el 60 % de los pacientes tratados con EGFR-TKI de primera y segunda generación. En casos con mutaciones atípicas en el gen *EGFR*, la respuesta al tratamiento puede variar con respecto a las mutaciones clásicas. Por ejemplo, las inserciones en el exón 20 suelen estar relacionadas con la resistencia a la terapia estándar con EGFR-TKI, aunque la respuesta puede ser heterogénea [12], [13].

## Metodologías para la caracterización molecular del gen *EGFR*

Las técnicas empleadas para la detección de mutaciones en el gen *EGFR* en el ctDNA se pueden clasificar en dos grupos: técnicas dirigidas y técnicas no dirigidas (véase la Tabla 1). Entre las técnicas dirigidas, se encuentran las que emplean la reacción en cadena de la polimerasa (RT-PCR); el sistema de mutación refractaria a la amplificación (ARMS); y métodos de PCR digital (dPCR), como la PCR digital de gotas (ddPCR) y BEAMing (por ***b*** *eads*, ***e*** *mulsions*, ***a*** *mplification*, *and* ***m*** *agnetics*). Aunque dichos métodos tienen una excelente especifidad, estos presentan algunas limitaciones a la hora de detectar deleciones y mutaciones puntuales predefinidas en su diseño.

**Tabla 1:** Listado de métodos empleados para el análisis molecular de *EGFR* en plasma (ctDNA).

Método	Mutaciones estudiadas	LoD	CE-IVD	FDA	Ref.
**Estrategias dirigidas**					

RT-PCR/ARMS					
EGFR mutation test v2 (Roche)	47 Mutaciones en: exones 18, 19, 20, 21 y T790M	25–100 copias/mL	Sí	Sí	[14]
Idylla™ ctEGFR mutation assay (Biocartis)	49 Mutaciones en: exones 18, 19, 20, 21 y T790M	<5 %	Sí	No	[15]
Therascreen EGFR plasma RGQ PCR kit (Qiagen)	29 mutaciones: deleciones exón 19, L858R, T790M	0,81-17,5 %	Sí	No	[16]
ctDNA EGFR mutation detection kit (entroGen)	58 Mutaciones en: exones 18, 19, 20, 21 y T790M + C797S	0,1–0,5 %	Sí	No	[17]
Easy EGFR (Diatech Pharmacogenetics)	T790M and C797S	<0,5 %	Sí	No	[18]
AmoyDx® EGFR mutation detection test (Amoy Diagnostics)	29 Mutaciones en: exones 18, 19, 20, 21 y T790M	1 %	Sí	No	[19]
The SuperARMS EGFR mutation detection kit (Amoy Diagnostics)	31 Mutaciones en: exones 18, 19, 20, 21 y T790M	0,2–0,8 %	Sí	No	[20]
PANAMutyper-R-EGFR (Panagene)	29 Mutaciones en: exones 18, 19, 20, 21 y T790M	0,1–0,5 %	Sí	No	[21]
dPCR y beaming					
ddPCR custom assays (Bio-Rad)	L858R, T790M, C797S, deleciones en el exón 19	0,1–0,4 %	RUO	No	[22]
ddPCR EGFR Exon 19 deletion screening kit (Bio-Rad)	15 deleciones en el exón 19	<0,5 %	RUO	No	[23]
OncoBEAM EGFR kit v2 (Sysmex)	36 mutaciones, incluidas T790M y C797S	0,1 %	RUO	No	[24]
Estrategias no dirigidas^a^					
Oncomine precision assay (OPA) (Thermo Fisher Scientific)	Regiones críticas del ADN, CNV y fusiones intragénicas	0,1–0,2 %	RUO	No	[25]
Oncomine™ lung cfDNA assay (Thermo Fisher Scientific)	T790M, C797S, L858R, deleciones en el exón 19	0,1 %	RUO	No	[26]
AVENIO ctDNA Targeted/Expanded/Surveillance kit v2 (Roche)	SNV, Indel, CNV	0,5–1 %	RUO	No	[27]
TruSight Oncology 500 ctDNA v2 (Illumina)	Pequeñas variantes, CNV, fusiones de ADN	0,2–0,5 %	RUO	No	[28]
Guardant360 CDx (Guardant Health, Inc)	SNV, InDels, CNV	0,2–1,8 %	Sí	Sí	[29]
FoundationOne Liquid CDx (Foundation medicine, Roche)	Cobertura completa de exones (sustituciones, indels) e intrones (7, 15, 24–27)	0,4–0,8 %	Sí	Sí	[30]

ARMS, *amplification refractory mutation system* (sistema de amplificación por mutación refractaria); CE-IVD, *In Vitro Diagnostic Medical Device* (dispositivo médico de diagnóstico *in vitro*) (uso aprobado en la Unión Europea); CNV, variantes en el número de copias; ddPCR, PCR digital en gotas; dPCR, reacción en cadena de la polimerasa digital; FDA, U.S. Food and Drug Administration (uso aprobado para los EE.UU); Indel, mutación por inserción/deleción; LoD, límite de detección; RT-PCR, reacción en cadena de la polimerasa con transcripción inversa; RUO, solo para fines de investigación; SNV, variante de un solo nucleótido. Información extraída de las especificaciones del fabricante. ^a^En paneles de cobertura amplia, la información sobre los tipos de mutación se centra en el gen *EGFR*. Los rangos del LoD dependen de la cantidad de cfDNA (ADN libre circulante) introducida y del tipo de variante analizada.

Con respecto a la sensibilidad analítica, las técnicas ARMS y RT-PCR son conocidas por su capacidad para detectar mutaciones de manera rápida y precisa, con un límite de detección (LOD) que oscila entre el 0,1 y el 5 % en la mayoría de las pruebas comerciales. Estos métodos proporcionan resultados cualitativos y semicuantitativos. Por otro lado, el método dPCR ofrece una mejor sensibilidad, alcanzando un LOD de entre el 0,1 y el 1 %, al dividir la muestra en numerosas reacciones discretas. Este método minimiza la interferencia de fondo, mejorando la detección de mutaciones de baja frecuencia. A diferencia de los métodos anteriores, la prueba de dPCR directamente proporciona resultados cuantitativos, permitiendo así determinar de forma precisa la frecuencia alélica de variantes (*VAF*, por sus siglas en inglés).

La secuenciación de próxima generación (NGS, *Next-Generation Sequencing)* es una técnica no dirigida que permite realizar una caracterización genómica completa, facilitando la detección de una amplia variedad de mutaciones en *EGFR* y otros genes relevantes, proporcionando resultados cuantitativos. La NGS también permite identificar nuevas mutaciones no detectadas en las pruebas dirigidas. Aunque la NGS tradicionalmente ha presentado ciertas limitaciones, como una menor sensibilidad y especifidad debido a las tasas de error inherentes a la ADN polimerasa y a los procesos de secuenciación, algunos avances, como la secuenciación profunda, los códigos de barras moleculares *(molecular barcoding)* y los algoritmos de corrección de errores, han mejorado notablemente su rendimiento. Gracias a estos avances, se ha logrado alcanzar niveles de sensibilidad sin precedentes (LOD de entre el 0,1 y el 0,5 %).

En cuanto al tiempo de respuesta (TDR), la exhaustividad de los resultados de la NGS conlleva un mayor TDR, ya que precisa la realización de complejos análisis computacionales, frente a las técnicas dirigidas como la RT-PCR o la dPCR. Aunque el TDR de los métodos de NGS es de entre una y dos semanas, Idylla y otros métodos de PCR presentan un TDR de aproximadamente siete días [31], [32], [33]. En resumen, aunque las técnicas dirigidas son muy eficaces en la detección de mutaciones específicas, con límites de detección bajos y un rápido TDR, la NGS proporciona una visión más completa del panorama mutacional.

Según el informe de 2022 de la Red Europea de Calidad Molecular (EMQN), la mayoría de los laboratorios aplica técnicas dirigidas, siendo la RT-PCR la técnica más extendida (51 %). De todas ellas, la prueba con mayor popularidad es la Cobas® EGFR Mutation Test v2 (Roche), empleada por el 27 % de los laboratorios. Esta fue la primera prueba de BL aprobada por la U.S. Food and Drug Administration (FDA), en 2016. Por otro lado, los laboratorios también han integrado técnicas de NGS, siendo Oncomine™ Lung cfDNA Assay (Thermo Fisher Scientific) el panel de mayor aceptación [34].

Los rápidos avances logrados en las tecnologías de NGS en los últimos años han reducido significativamente los costes de secuenciación, habiendo mejorado a su vez su exactitud. Así mismo, a medida que va creciendo la lista de alteraciones génicas susceptibles de intervención *(actionable gene mutations)* con tratamientos aprobados por la FDA (*EGFR*, *ALK*, *ROS1*, *BRAF*, *RET*, *MET*, *NTRK*, *KRAS*, *HER2*), también se van intensificando las recomendaciones de emplear paneles más amplios de NGS. Estos paneles no solo presentan una mejor relación coste-beneficio que las pruebas de secuenciación de un solo gen en muestras de tejido, sino que también están ganando terreno en el análisis del plasma. La Asociación Internacional para el Estudio del Cáncer de Pulmón (IASLC) y la Sociedad Europea de Oncología Médica (ESMO) recomiendan la realización de un análisis amplio de ctDNA plasmático mediante NGS, en casos de CPNM avanzado [34], [35], [36]. En regiones con una prevalencia elevada de mutaciones en *EGFR*, el primer paso a la hora de realizar el análisis molecular seguirá siendo llevar a cabo un análisis dirigido. Sin embargo, si se obtiene un resultado negativo en el análisis de un único gen, es aconsejable realizar pruebas en serie para identificar otros biomarcadores de interés clínico.

## Comparación entre el ctDNA plasmático y las muestras de tejido para el análisis molecular del gen *EGFR*

Hasta la fecha, el tejido tumoral ha sido la piedra angular del análisis molecular de *EGFR*. Sin embargo, presenta algunas limitaciones inherentes, como su naturaleza invasiva y las limitaciones asociadas a la obtención de biopsias de localizaciones de difícil acceso o en pacientes con patologías complejas. Así mismo, las muestras de tejido tumoral pueden aportar ADN insuficiente o degradado, complicando su análisis. El análisis del gen *EGFR* en ctDNA extraido del plasma ha mostrado un elevado nivel de concordancia con las muestras de tejido, lo que permite solventar algunas de estas limitaciones. El análisis de ctDNA en plasma presenta diversas ventajas: es mínimamente invasivo, presenta una elevada concordancia con los resultados obtenidos en tejido, ofrece un TDR rápido, y permite repetir posteriormente la prueba, proporcionando una representación más completa de la heterogeneidad tumoral y la evolución clonal. Por otra parte, presenta otras limitaciones, como el riesgo de obtener falsos negativos y su elevado coste cuando se emplea paralelamente al análisis de tejido [35].

Los estudios en los que se ha comparado el análisis de ctDNA en plasma con el análisis de tejido tumoral han revelado un elevado nivel de concordancia en relación a las mutaciones de *EGFR*, así como en otros biomarcadores recomendados por las guías de práctica clínica [37], [38]. González de Aledo-Castillo et al. obtuvieron una concordancia del 95,2 % con el análisis de tejido tumoral para el análisis de cfDNA plasmático mediante la prueba Cobas® EGFR en pacientes con CPNM en estadio III y IV. En su estudio prospectivo, no se dispuso de tejido tumoral accesible para la caracterización molecular en el 39,9 % de los pacientes. En este escenario, el análisis de *EGFR* en plasma permitió iniciar la terapia en el 34,8 % de estos pacientes, lo que evidencia la utilidad del ctDNA cuando no resulta viable obtener una biopsia de tejido [14].

En cuanto al rendimiento analítico, el análisis de ctDNA en plasma suele demostrar una elevada especifidad en las diferentes plataformas (>96 %). Por otro lado, su sensibilidad varía considerablemente (58–100 %) en función de la plataforma y las mutaciones estudiadas [35]. Por ejemplo, las mutaciones T790M se suelen detectar a frecuencias bajas en el ctDNA plasmático, frente a las mutaciones sensibilizantes primarias (L858R o deleciones en el exón 19), lo que reduce su sensibilidad (41–90,5 %) [35]. En un meta-análisis reciente se obtuvo una sensibilidad combinada del 68 % (95 % CI=60–75 %) y una especifidad del 98 % [95 % CI=95–99 %) [39].

Otra ventaja notable del análisis de ctDNA es el menor TDR, si lo comparamos con el genotipado de tejido, con una mediana de TDR de 12 días (intervalo 1–54 días) [40], [41]. En general, el análisis de ctDNA plasmático permite disponer de los resultados de la prueba con anterioridad a la primera consulta con el paciente (85 % frente al 9 %, p<00,001), reduciendo de este modo el tiempo transcurrido hasta iniciar el tratamiento en pacientes con CPNM avanzado [42].

El análisis de ctDNA en plasma también ofrece la posibilidad de realizar un seguimiento longitudinal de la respuesta al tratamiento y de la progresión de la enfermedad en pacientes tratados con EGFR-TKI [43]. La repetición de biopsias de tejido puede conllevar cierto riesgo, debido al estado clínico del paciente o a la localización del tumor, por lo que el análisis del plasma representa una alternativa más segura a la hora de evaluar el estado mutacional de *EGFR* durante el tratamiento [44]. Además, el análisis de ctDNA puede proporcionar información muy valiosa sobre mecanismos de resistencia como la amplificación de MET, revelando la heterogeneidad tumoral y la evolución clonal en los casos de resistencia al osimertinib con mutaciones de *EGFR* (ensayo AURA3, NCT02151981) [45], [46]. Sin embargo, cabe señalar que el análisis de ctDNA puede no identificar la totalidad de los mecanismos de resistencia, como las transiciones histológicas, que requieren de un análisis morfológico de tejido.

Los falsos negativos siguen siendo el talón de Aquiles del análisis de ctDNA, por lo que aún es necesario realizar una biopsia de tejido cuando las pruebas de BL arrojan resultados negativos. La sensibilidad puede variar en función de una serie de factores analíticos y biológicos. Al igual que otros marcadores circulantes, como los marcadores tumorales séricos (MTS), las características biológicas del tumor, como el estadio, la carga tumoral y la vascularización, pueden influir en la cantidad de ctDNA liberado en la circulación, lo que afecta a la sensibilidad de la prueba. Entre el 15 y el 32 % de los pacientes con CPNM presentan tumores con una diseminación de ctDNA baja [47], y su sensibilidad se ve aún más reducida en el CPNM en estadios tempranos, siendo esta inferior al 30 % [48]. Se pueden obtener falsos negativos debido a las limitaciones de la técnica, lo que subraya la necesidad de centrarnos en métodos de elevada sensibilidad como la NGS. Algunos estudios han explorado otras matrices alternativas, como el fluido de lavado bronquial, que ha mostrado una mayor sensibilidad para la detección de mutaciones de *EGFR* que el plasma [49], [50]. Los falsos positivos son poco frecuentes, aunque estos pueden ocurrir, especialmente cuando se emplean métodos de amplio espectro como la NGS, en la que se analizan múltiples genes. Los falsos positivos se pueden producir por la presencia de variantes germinales o de fuentes no tumorales como la hematopoyesis clonal de potencial indeterminado (CHIP, por sus siglas en inglés). Aunque las variantes CHIP pueden influir en las mutaciones en genes como *TP53* y *KRAS*, estas no tienen un impacto significativo en el análisis mutacional del *EGFR* [51].

En conclusión, el análisis molecular del gen *EGFR* en ctDNA plasmático ofrece ventajas significativas, como el hecho de ser un método no invasivo y accesible que permite realizar un seguimiento de la enfermedad. Sin embargo, las limitaciones de esta técnica impiden que esta pueda sustituir al genotipado de tejido, por lo que esta debe emplearse como una herramienta complementaria en la toma de decisiones terapéuticas en el CPNM avanzado.

## Factores preanalíticos y postanalíticos

La fase preanalítica influye de manera fundamental en los resultados del análisis de cfDNA. Resulta esencial ajustarse a las directrices establecidas para la obtención, el manejo y el transporte de las muestras, así como para la extracción de cfDNA, con el fin de optimizar su estandarización y fiabilidad [52]. Entre los principales factores que afectan a la calidad del análisis de cfDNA se encuentran: 1) el contenido heterogéneo de la sangre, que complica el aislamiento de cfDNA; 2) la degradación enzimática y la coagulación; 3) la inestabilidad del ADN libre en los entornos biológicos; 4) la contaminación del cfDNA por la presencia de ADN genómico (gDNA); y 5) la dificultad a la hora de detectar la pequeña fracción específica de ctDNA dentro del conjunto de cfDNA.

Con respecto al aislamiento del cfDNA, es preferible emplear el plasma al suero, con el fin de evitar su posible contaminación por la presencia de gDNA procedente de los leucocitos alterados durante la coagulación [53]. Se debe extraer la sangre en tubos que contengan un estabilizador celular (Streck^®^) o EDTA, que es el anticoagulante recomendado. Si se utiliza un tubo EDTA, el plasma se debe aislar en un margen de cuatro horas [53]. Cuando es necesario transportar las muestras a diferentes centros, es preferible emplear tubos comerciales con estabilizadores celulares, ya que estos previenen la lisis de las células sanguíneas, permitiendo ampliar los tiempos de procesamiento a varios días, a temperatura ambiente. Se recomienda centrifugar el plasma en dos pasos: en primer lugar, se realiza una centrifugación a baja velocidad para concentrar las células sanguíneas, para posteriormente realizar una centrifugación a alta velocidad para eliminar los desechos celulares. En cuanto al método de extracción, es preferible emplear kits automáticos ya preparados para sus aplicaciones más frecuentes [52]. También resulta aconsejable realizar un control de la calidad del extracto de cfDNA para comprobar tanto la cantidad de cfDNA como los niveles de fragmentación [54].

En la fase postanalítica, la interpretación y emisión de resultados sobre las variantes de *EGFR* en el ctDNA deben realizarse con ajuste a los criterios estandarizados para la interpretación de variantes somáticas [55], [56]. También es de gran importancia tener en cuenta las características únicas del ctDNA y ajustarse a las directrices específicas para el análisis de ctDNA [37], [57], con el objeto de garantizar una detección exacta y una emisión de los resultados sobre las mutaciones en el gen *EGFR* pertinentes para el CPNM.

Los informes moleculares sobre cfDNA deben ser claros y concisos, haciendo énfasis en la información clínicamente relevante. Las alteraciones genéticas se deben describir empleando la nomenclatura estandarizada de la guía de la Human Genome Variation Society (HGVS) (http://varnomen.hgvs.org). Así mismo, los informes deben ser redactados empleando términos simplificados y coloquiales, con el fin de mejorar su claridad e inteligibilidad. Por ejemplo, una alteración en el gen *EGFR* se debe mencionar como “c.2369C>T (p.Thr790Met)” y ser descrita además como la “mutación T790M”. En los informes, se deben ofrecer detalles que faciliten la toma de decisiones clínicas, haciendo hincapié en la significación clínica de las variantes identificadas, con respecto a las terapias aprobadas por la FDA y/o la EMA. Por otro lado, al final del documento, se debe incluir información metodológica que especifique las limitaciones de las alteraciones analizadas, las frecuencias alélicas, la cobertura y otros datos técnicos pertinentes. En la emisión de resultados, se debe hacer mención de la posibilidad de que existan discrepancias entre el análisis del tumor, especialmente en situaciones donde no se haya detectado una variante en el ctDNA plasmático [37]. Teniendo en cuenta la posibilidad de que se produzcan falsos negativos, los resultados sobre *EGFR* no deben ser informados como meramente negativos, sino que estos deben ser descritos como “no informativo” o “no detectado”. Esta terminología refleja la posibilidad de que el tejido presente una mutación en *EGFR* que no se haya detectado en el plasma, debido a factores biológicos o a limitaciones técnicas.

Finalmente, los comités moleculares oncológicos desempeñan un papel crucial a la hora de interpretar casos complejos, ya que estos comités están formados por expertos procedentes de distintas disciplinas. Estos comités garantizan la realización de una evaluación exhaustiva de los hallazgos moleculares, donde los profesionales de laboratorio cobran una especial relevancia, ya que garantizan la correcta interpretación de las alteraciones moleculares, especialmente ante la presencia de variantes de significado incierto o de resultados contradictorios.

## Utilidad clínica y recomendaciones en los distintos escenarios clínicos

Las guías nacionales e internacionales recomiendan el genotipado de ctDNA1 como sustituto del genotipado de tejido en el análisis molecular de *EGFR* y otros biomarcadores relevantes en el CPNM avanzado [58], [59]. Esto es aconsejable cuando no se dispone de muestras de tejido, ya sea para realizar el diagnóstico inicial o para realizar un seguimiento del desarrollo de resistencia al tratamiento. Los algoritmos de decisión elaborados por organizaciones como la IASLC ayudan a elegir entre realizar un análisis de tejido o de plasma. La IASLC recomienda aplicar la estrategia de “primero el plasma” cuando no se dispone de una muestra de tejido; una “estrategia complementaria” cuando se dispone de tejido pero este resulta insuficiente o de idoneidad dudosa para el genotipado; y una “estrategia secuencial” cuando el genotipado de tejido es incompleto [35].

Por el contrario, el análisis de tejido sigue siendo el método de elección para el CPNM en estadios iniciales (estadios I–III), situación en la que se encuentran entre el 25 y el 30 % de los pacientes. Osimertinib, un EGFR-TKI de tercera generación, fue aprobado por la FDA en el año 2020 para reducir la tasa de recurrencia post cirugía en pacientes con CPNM en estadios de IB a IIIA con mutaciones activadoras en *EGFR*, en base a los resultados del ensayo ADAURA, que mostraron una mejora significativa en la supervivencia libre de enfermedad (SLE) [60]. En un contexto neoadyuvante, las terapias anti-EGFR pueden reducir el tamaño del tumor y mejorar las tasas de resección. Sin embargo, los estudios en curso, como NeoADAURA, serán fundamentales para seguir mejorando las estrategias de tratamiento en este contexto.

En el CPNM en fases tempranas, no se recomienda analizar sistemáticamente el ctDNA, ya que su utilidad clínica sigue en estudio. Su potencial radica en su posible utilidad como biomarcador pronóstico para evaluar el riesgo de recurrencia. Un gran número de pacientes experimentan recidiva tras la cirugía, y el seguimiento del cfDNA tras la misma ofrece la oportunidad de una detección precoz, permitiendo una intervención rápida o un seguimiento más estrecho, antes de que aparezcan los primeros signos radiológicos de recidiva. En los pacientes con NSCLC-*EGFR*+, la presencia o ausencia de ctDNA con anterioridad y posterioridad a la cirugía constituye un sólido indicador pronóstico, estando la negatividad o eliminación del ctDNA relacionadas con una mejor SLE. Cabe mencionar que la mayoría de los pacientes con enfermedad residual mínima indetectable mediante el seguimiento longitudinal de ctDNA no experimentaron ninguna recidiva [61].

En cuanto al cribado, es imperativo contar con métodos de cribado no invasivos que mejoren la detección precoz del CP en poblaciones de alto riesgo, así como en la población general. Los estudios realizados sobre biomarcadores circulantes, entre los que se incluyen proteínas, autoanticuerpos, perfiles de expresión génica, y microRNA, han arrojado resultados prometedores para la detección precoz y la distinción del cáncer de los nódulos no cancerosos [62]. Actualmente, ninguna de estas estrategias ha sido aprobada para su uso clínico. Los análisis sanguíneos basados en las técnicas de BL son capaces de identificar diversos tipos de cáncer resecable quirúrgicamente mediante la detección de mutaciones en cfDNA en oncogenes clave, como *EGFR*, y mediante el análisis de proteínas circulantes. Sin embargo, su sensibilidad para detectar dichas alteraciones en el cáncer de pulmón (CP) en estadios iniciales sigue siendo relativamente baja [63].

La utilización de diferentes biomarcadores y técnicas en combinación con las características clínicas es la estrategia con mayor probabilidad de mejorar la sensibilidad y especifidad de la prueba. Un estudio reciente demostró que al integrar el análisis de fragmentación del cfDNA con los factores de riesgo clínico y la concentración de MTS (CEA) mejora significativamente la detección en la BL en casos de cáncer de diferentes estadios y subtipos, siendo del 91 % en los estadios I/II y del 96 % en los estadios III/IV, con una especifidad del 80 % [64].

## Correlación entre el estadio de *EGFR* y los marcadores tumorales séricos

El circuloma tumoral consiste en el análisis de biomarcadores genéticos (cfDNA) además de otros biomarcadores derivados del tumor, como las proteínas y otras moléculas liberadas en el torrente sanguíneo. Esta nueva estrategia multiómica está suscitando interés en el campo de la oncología de precisión [65]. Los MTS son glucoproteínas segregadas en la circulación por las células tumorales o el microambiente tumoral. Aunque los MTS se suelen medir en el laboratorio clínico para el manejo del neoplasma epitelial, su utilidad clínica en el CP sigue siendo objeto de debate. Algunos estudios prospectivos muestran que la combinación de seis MTS (CEA, CYFRA 21.1, CA 15.3, SCC, NSE, y ProGRP) con algunos parámetros clínicos es la estrategia más exacta para detectar el CP en pacientes sintomáticos [66]. Así mismo, hallazgos recientes indican la posible utilidad de integrar los MTS y el análisis de ctDNA para el diagnóstico precoz del cáncer de pulmón [63], [67]. La combinación de MTS y ctDNA posee valor predictivo, estando los niveles elevados relacionados con un peor pronóstico y una menor supervivencia libre de progresión tras la terapia con EGFR-TKI [68]. Sin embargo, aún no disponemos de suficiente evidencia científica que respalde la integración de alguno de estos biomarcadores sanguíneos en las guías de práctica clínica para el diagnóstico del CP.

Con respecto al perfil molecular, se han relacionados varias MTS con el estado mutacional del gen *EGFR* u otras alteraciones moleculares, tal como se muestra en la Tabla 2. En la mayoría de los estudios, se ha observado una relación entre los niveles elevados de CEA y CA 15–3, y/o niveles reducidos de CYFRA 21–1 y SCC, y la presencia de mutaciones en *EGFR* [69], [70], [71], [72], [73], [74], [75]. Estos hallazgos coinciden con la evidencia existente sobre el valor de algunos marcadores concretos para predecir algunos perfiles histológicos: CEA y CA 15–3 para el adenocarcinoma, donde las mutaciones en *EGFR* son más prevalentes, y CYFRA 21–1 y SCC para el carcinoma de células escamosas [66]. Por el contrario, en otros estudios no se han observado diferencias significativas entre el estado del gen *EGFR* y la presencia de STM [76], [77], [78]; concretamente, en pacientes con enfermedad en estadio inicial, cuya expresión en la circulación y, por tanto, la sensibilidad, son bajas [70]. Durante el seguimiento de la terapia con EGFR-TKI, las STM dinámicas podrían predecir la eficacia del tratamiento [79], así como la presencia de la mutación secundaria T790M.

**Tabla 2:** Resumen de los estudios en los que se evalúa la relación entre los marcadores tumorales séricos y el estado molecular de *EGFR*.

Título	Cohorte	MTS	Principales conclusiones	Año	Ref
*Distinction of ALK fusion gene and EGFR mutation-positive lung cancer with tumor markers* *** *** *** ***	306 CP Estadio III/IV 43,8 % *ALK*+ 56,2 % *EGFR*+	CEA CYFRA 21-1	Se observó una mayor proporción de pacientes *ALK*+ con CYFRA 21-1 positivo ratios. atios más elevados de CYFRA 21-1:CEA en pacientes *ALK*+ en comparación con los pacientes *EGFR*+.	2024	[69]
*The predictive value of serum tumor markers for EGFR mutation in non-small cell lung cancer patients with non-stage IA* *** ***	6711 CPNM Estadio IA y superiores 57,9 % *EGFR*+	CEA CYFRA 21-1 SCC	Asociaciones significativas con las mutaciones de *EGFR* en estadios distintos al IA. No se hallaron MTS predictores en el estadio IA. La combinación de MTS con factores clínicos predice eficazmente la presencia de mutaciones en *EGFR*.	2024	[70]
*Dynamic monitoring serum tumor markers to predict molecular features of EGFR- mutated CP during targeted therapy* *** ***	303 CP Estadios III-IV 43 % *EGFR*+	CEA CYFRA 21-1 NSE CA125 CA153 SCC ctDNA	Mutaciones en *EGFR*: asociadas significativamente con el género femenino, niveles alterados de CEA y CA153, y niveles normales de SCC. Mutación T790M: Más común en pacientes con niveles alterados de CEA. Niveles basales de MTS y sus oscilaciones: pueden indicar la presencia de la mutación secundaria T790M.	2022	[71]
*Establishment and evaluation of EGFR Mutation Prediction Model Based on Tumor Markers and CT Features in NSCLC* *** ***	148 CPNM Estadios I-IV 51 % *EGFR*+	CEA CYFRA 21-1 NSE CA 125 CA 19.9	Factores predictivos de la presencia de mutaciones en *EGFR*: no fumadores, nivel elevado de CEA y bajo de CYFRA 21-1. Niveles de CYFRA 21-1: más altos en el grupo *wild-type.* Niveles de CA 19.9: más altos en el grupo con mutaciones en *EGFR*.	2022	[72]
*Value of serum tumor markers for predicting EGFR mutations and positive ALK expression in 1089 Chinese non-small-cell lung cancer patients: A retrospective analysis.* *** ***	1089 CPNM Estado mutacional de *EGFR* 50,1 % *EGFR*+ (1088 evaluados)	CEA CA 125 SCC CYFRA 21-1 FERR	Mutaciones en *EGFR* asociadas a ADC, nunca fumador, y negatividad para CA 125, SCC, FERR, CYFRA 21-1. El análisis multivariante demostró que ADC, la ausencia de hábito tabáquico, y la negatividad para CA 125 y SCC son factores predictores de la presencia de mutaciones en *EGFR.*	2020	[73]
*Value of serum tumor markers for predicting EGFR mutations in non-small cell lung cancer patients* *** ***	143 CPNM 44,06 % *EGFR+*	CEA CYFRA 21-1 NSE SCC ProGRP	Los MTS están asociados al estado mutado de *EGFR* y podrían integrarse con otros factores clínicos para facilitar la clasificación del estado mutacional de *EGFR* entre los pacientes con CPNM. El análisis de regresión logística univariante mostró una relación estadísticamente significativa entre el género, el hábito tabáquico, el tipo histológico, SCC, proGRP y la presencia de mutaciones en *EGFR*.	2020	[80]
*Combining PET/CT with serum tumor markers to improve the evaluation of histological type of suspicious lung cancers* *** ***	201 CP Estadios I-IV 50 % *EGFR*+ (16 evaluados)	CEA CYFRA 21-1 NSE SCC	No se observaron diferencias significativas entre los diferentes estados mutacionales de *EGFR* en cuanto a SUVmax, CEA, CYFRA 21-1, SCC-Ag o NSE.	2017	[76]
*Predictive and Prognostic Value of CYFRA 21-1 for advanced non-small cell lung cancer treated with EGFR-TKIs* *** ***	95 CPNM Estadios IIB-IV 57 % *EGFR*+	CEA CYFRA 21-1	El nivel sérico de CYFRA 21-1 puede ser un factor predictivo en pacientes con CPNM tratados con EGFR-TKIs, independientemente del estado mutacional de *EGFR*. Los niveles elevados de CYFRA 21-1 en suero se asociaron con una menor SLP y SG en pacientes con CPNM tratados con EGFR-TKI.	2017	[79]
*Correlation between EGFR gene mutation, cytologic tumor markers, 18F-FDG uptake in non-small cell lung cancer* *** ***	61 CPNM Estadios I-IV 49,1 % *EGFR*+	Serum and cytologic CEA CYFRA 21-1 SCC	No se observaron diferencias significativas en los niveles de MTS entre los pacientes con *EGFR wild-type* y aquellos con *EGFR* mutado Los niveles de c-CYFRA fueron significativamente superiores en los pacientes con mutaciones en *EGFR*, comparados con aquellos con *EGFR* wild-type.	2016	[77]
*Predictive and prognostic value of preoperative serum tumor markers is EGFR mutation-specific in resectable non-small-cell lung cancer* *** ***	1016 CPNM I-IIIA 25 % *EGFR*+ (979 evaluados)	CEA CYFRA 21-1 SCC NSE	No se observaron diferencias en los niveles de CEA o CYFRA21-1 entre los pacientes con adenocarcinoma *EGFR* positivo y aquellos *wild-type*. CYFRA 21-1 sirve como marcador predictivo y pronóstico en los pacientes con adenocarcinoma resecable y mutaciones en *EGFR*, especialmente en los grupos con deleción del exón 19 o mutación L858R. CEA es un factor predictivo y pronóstico independiente solo en los pacientes con adenocarcinoma con *EGFR wild-type* o con la mutación L858R.	2015	[78]
*Monitoring of carcinoembryonic antigen levels is predictive of EGFR mutations and efficacy of EGFR-TKI in patients with lung adenocarcinoma* *** ***	70 ADC Estadio IIA-IV 62,9 % *EGFR*+	CEA	Un nivel elevado de CEA se asociacia de forma independiente con la presencia de mutaciones en el gen *EGFR.* Los diferentes patrones de variación en los niveles de CEA podrían ayudarnos a predecir la eficacia de EGFR-TKI en pacientes que presentan una mutación en *EGFR* en tan solo un mes de terapia con TKI.	2014	[74]
*Correlation between EGFR mutations and serum tumor markers in lung adenocarcinoma patients * *** *** *** ***	70 ADC Estadios I-IV 38,6 % *EGFR*+	CEA CA 242	Existe una asociación entre los niveles séricos de CEA y CA 242 y la presencia de mutaciones en *EGFR*.	2013	[75]

En los estudios en los que no se proporciona información sobre el estadio de la enfermedad, este no se detalla en la columna de cohorte. ADC, adenocarcinoma; ALK, quinasa del linfoma anaplásico; CEA, antígeno carcinoembrionario; CA125, antígeno de carbohidrato 125; CA153, antígeno de carbohidrato 153; CA 242, antígeno de carbohidrato 242; CYFRA 21-1, fragmentos de citoqueratina-19; c-CYFRA 21-1, CYFRA 21-1 citológica; ctDNA, ADN tumoral circulante; EGFR, receptor del factor de crecimiento epidérmico; FERR: ferritina; CP, cáncer de pulmón; NSE, enolasa específica neuronal; SG, supervivencia global; SLP, supervivencia libre de progresión; SCC, antígeno del carcinoma de células escamosas; MTS, marcadores tumorales séricos; TKI, inhibidor de la tirosina quinasa.

Toda esta evidencia indica que las STM se podrían emplear de manera conjunta con otros factores clínicos para predecir el estado mutacional del gen *EGFR*, por lo que el seguimiento combinado del ctDNA + STM podría ofrecer información sobre la respuesta a la terapia, mejorando la selección de las terapias personalizadas. Sin embargo, los diversos estudios realizados hasta la fecha han arrojado resultados discordantes, lo que pone de manifiesto la necesidad de seguir investigando su utilidad clínica.

Los avances realizados en el campo de la inteligencia artificial y el aprendizaje automático podrían ser de utilidad a la hora de diseñar modelos basados en una combinación eficaz de múltiples marcadores, en los que se integren datos clínicos y de imagen. Esta estrategia facilitará el análisis de grandes volúmenes de datos clínicos y de imagen para identificar patrones con capacidad para predecir la presencia y progresión del cáncer [81], [82]. Estos avances permitirán desarrollar herramientas de apoyo a la toma de decisiones clínicas y ofrecerán la oportunidad de desarrollar tratamientos oncológicos personalizados más efectivos.

## Conclusiones

Esta revisión ofrece una serie de directrices prácticas para integrar el análisis de ctDNA en la clínica real. Las mutaciones en *EGFR* representan una diana terapéutica fundamental en el CPNM avanzado, y la limitada disponibilidad de muestras de tejido evidencia la necesidad de desarrollar métodos no invasivos, como el análisis de ctDNA en plasma. Las actuales guías de práctica clínica recomiendan sustituir el genotipado de tejido por el genotipado de ctDNA cuando no se dispone de muestras de tejido, así como emplear algoritmos de decisión para elegir entre realizar un análisis de plasma o de tejido.

El laboratorio clínico desempeña un papel fundamental a la hora de garantizar la fiabilidad del análisis molecular del gen *EGFR* en ctDNA tanto en la fase preanalítica como postanalítica. Aunque los métodos dirigidos siguen siendo la técnica más frecuente en la práctica clínica por su solidez y rápido TDR, los métodos de NGS están ganando terreno, al proporcionar un perfil mutacional más completo. Este cambio viene dado por la constante evolución de las terapias dirigidas y la creciente necesidad de obtener análisis más exhaustivos que permitan identificar los mecanismos de resistencia.

Más allá de su utilidad en el CPNM avanzado, se están investigando las posibles aplicaciones del análisis de ctDNA en los estadios iniciales de esta enfermedad. La posible aplicación de las terapias adyuvantes y neoadyuvantes con EGFR-TKI hace necesario disponer de métodos no invasivos para analizar el estado mutacional del gen *EGFR* en los estadios iniciales del CPNM y optimizar la selección de las terapias dirigidas. Aunque se ha demostrado el valor pronóstico del análisis de ctDNA tras la cirugía, su baja sensibilidad limita su posible uso en los estadios iniciales y el cribado del CP. El análisis del circuloma tumoral, en el que se incluyen las alteraciones moleculares, la fragmentación del cfDNA y las proteínas o biomoléculas liberadas por las células tumorales en la circulación, es un método no invasivo muy prometedor. Esta estrategia podría mejorar la sensibilidad y precisión de las pruebas, permitiendo la detección precoz y la optimización de los tratamientos personalizados.
